# Obtaining and doping of InAs-QD/GaAs(001) nanostructures by ion beam sputtering

**DOI:** 10.3762/bjnano.8.2

**Published:** 2017-01-03

**Authors:** Sergei N Chebotarev, Alexander S Pashchenko, Leonid S Lunin, Elena N Zhivotova, Georgy A Erimeev, Marina L Lunina

**Affiliations:** 1 Department of Physics and Electronics, Platov South-Russian State Polytechnic University (NPI), 346428, 132, Prosveshchenia str., Novocherkassk, Russia; 2 Department of Nanotechnology and Solar Energy, Southern Scientific Center of Russian Academy of Sciences, 344006, 41, Chekhov Avenue, Rostov-on-Don, Russia

**Keywords:** 3D growth, doping, ion-beam sputtering, photoluminescence, quantum dots

## Abstract

The features of InAs quantum dots obtained on GaAs(001) single-crystal substrates by ion-beam sputtering were investigated. It has been shown that in the range of ion energies of 150 to 200 eV at a temperature of 500 °C and a beam current of 120 µA InAs quantum dots with average dimensions below 15 nm and a surface density of 10^11^ cm^−2^ are formed. The technique of controlled doping of InAs/GaAs nanostructures using a SnTe solid-state source was proposed. It has been established that a maximum donor concentration of 8.7·10^18^ cm^−3^ in the GaAs spacer layer is reached at an evaporation temperature of 415 °С. At the same time, impurity accumulation in the growth direction was observed. We have shown that increasing the impurity doping of the GaAs barrier layer increases the intensity of photoluminescence peaks of the ground state and the first excited state of the InAs quantum dots.

## Introduction

Main interests of inorganic nanotechnology science are the study of semiconducting [1], magnetic [2] and superconducting [3] nanomaterials. Among them, InAs/GaAs nanostructured materials have a considerable application potential in lasers [4], photonic devices [5], photoelectric converters based on multilayer heterostructures [6] and intermediate band devices [7].

Molecular beam epitaxy [8] and vapour phase epitaxy [9] are commonly used and well-understood techniques for obtaining such nanostructures. Besides the mentioned methods, classic growth methods such as liquid phase epitaxy [10], laser beam sputtering [11], electron beam sputtering [12] and ion beam sputtering [13] are being actively adapted for the growth of nanomaterials with quantum dots (QDs).

Ion beam sputtering of germanium films was firstly carried out by Krikorian and Sneed [14]. Their work demonstrated a significant potential of the technique and became a starting point of its development. Ion beam homoepitaxy of silicon on substrates with (001) crystallographic orientation was in parts investigated by Lee and Xue [15]. High-vacuum ion beam heteroepitaxy of nanometer-thick germanium films on silicon substrates was carried out by Alexandrov and co-workers [16]. They were the first to observe the self-assembly growth of germanium quantum-dot nanostructures.

Furthermore, ion beam sputtering was used for heteroepitaxy of Ge on GaAs substrates [17] and GaAs_1−_*_x_*P*_x_* on Si substrates [18]. In addition, the effect of ion beam bombardment of semiconductor surfaces is used at least for two applications. First, it is employed to form nanostructured patterns on semiconductor surfaces [19]. And second, this effect was applied to stimulate nucleation nanoislands by ion-assisted molecular beam epitaxy [20]. It allowed the reduction of size and size dispersion of QDs.

Earlier, we studied some features of crystallization of quantum-dimensional Ge/Si [21] and InAs/GaAs [22–23] heterostructures by ion-beam sputtering. The features of ion beam crystallization of silicon films [24] were partially investigated. Also, the morphology of Ge-QD/Si nanostructures [25] and photoluminescence of InAs-QD/GaAs nanostructures [26] were studied.

Above-mentioned publication [22] was the first experimental work in which we investigated the ion-beam crystallization of InAs quantum dots onto GaAs substrates. The growth conditions of the crystallization process were not optimized, so the produced InAs nanoclusters had planar dimensions from 20 to 100 nm. In [23] the GaAs and InAs sputtering yields in the ion energy range of 200–300 eV under an incidence angle of 30° were refined. We demonstrated that growth rates of up to 0.1 ML/s for InAs and 0.05 ML/s for GaAs could be attained. But in that work comprehensive research about the crystallization depending on temperature, energy and beam current was not performed. Neither did we focus on doping processes in our earlier articles.

The aim of the present study is to generalize features of crystallization and doping of InAs-QD/GaAs(001) quantum-dot nano-heterostructures grown by ion beam sputtering.

## Experimental

The samples were obtained by using an ion beam sputtering facility equipped with a vacuum chamber, a vacuum pump Varian DS 302, a turbomolecular pump Leybold Turbovac 340, an ion source KDC 40 and a cryotrap cooled with liquid nitrogen. The residual pressure in the chamber was 5·10^−7^ Pa. The ion current was measured by a Faraday cup of 1 mm diameter fixed on the target holder.

GaAs and InAs two-inch wafers having (001) crystallographic orientation were used as targets; the wafers were preliminary cleaned with argon ions. The wafers were isolated from the targets by molybdenum screens during cleaning. Ion etching was carried out at an energy of 180 eV and with etching rates less than 0.3 ML/s. It was shown in [27] that the incorporation of argon ions in the GaAs wafer was not observed at energies below 200 eV. This technique makes it possible to remove the oxide films containing adsorbed impurities.

The described way of cleaning the targets cannot be used for the pre-treatment of single-crystal substrates because of possible radiation damage. For this purpose, we used the procedure described in [28]. Pre-growth annealing of the prepared substrate in the vacuum chamber at a temperature of 560–580 °C allowed us to get rid of a protective oxide layer and the impurities accumulated in it.

**Figure 1:**
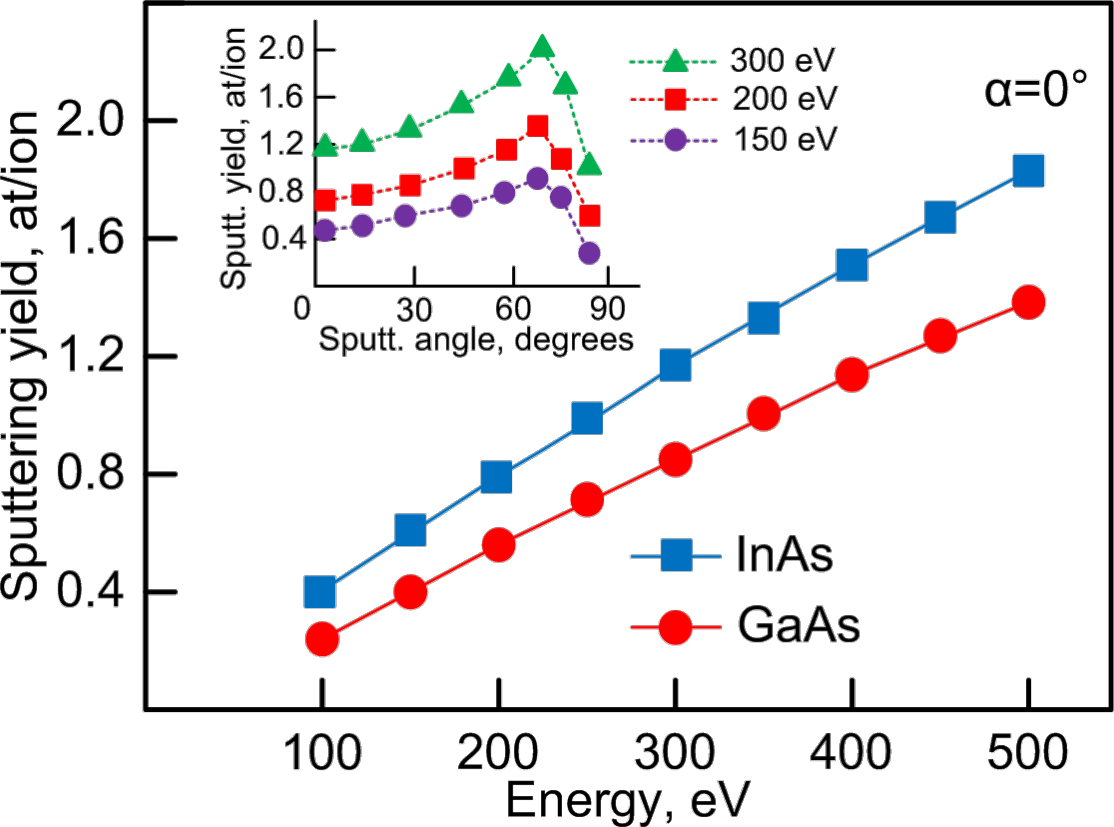
Energy dependence and angular dependence of sputtering coefficients.

The accelerating voltage determining the ion energy varied in the range from 100 to 500 V. The energy dependence of the sputtering yields was measured with a step of 50 eV. The experimental yields of GaAs and InAs are given in Figure 1. The ion energy of 100 eV is close to the threshold energies of the selected semiconductors. The usage of lower energies leads to uncontrolled sputtering. The usage of ion energies more than 500 eV is not required because of the high sputtering yields.

The obtained data on sputtering yields were used for the calculation of the deposition rate of InAs and GaAs. It should be noted that the thickness of one monolayer (1ML) of gallium arsenide and indium arsenide corresponds to a flux density of *f*_1МL_ = 6.26 × 10^14^ cm^−2^ and *f*_1МL_ = 5.45 × 10^14^ cm^−2^, respectively. These values were used for calibrating the deposition rate (ML/s) from the values of flux density (cm^−2^). Knowing the ion current density *j* measured by the Faraday cup and the experimentally found values of sputtering yield *Y*, the deposition rate can be calculated by the formula


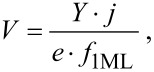


where *e* is the electron charge.

**Figure 2:**
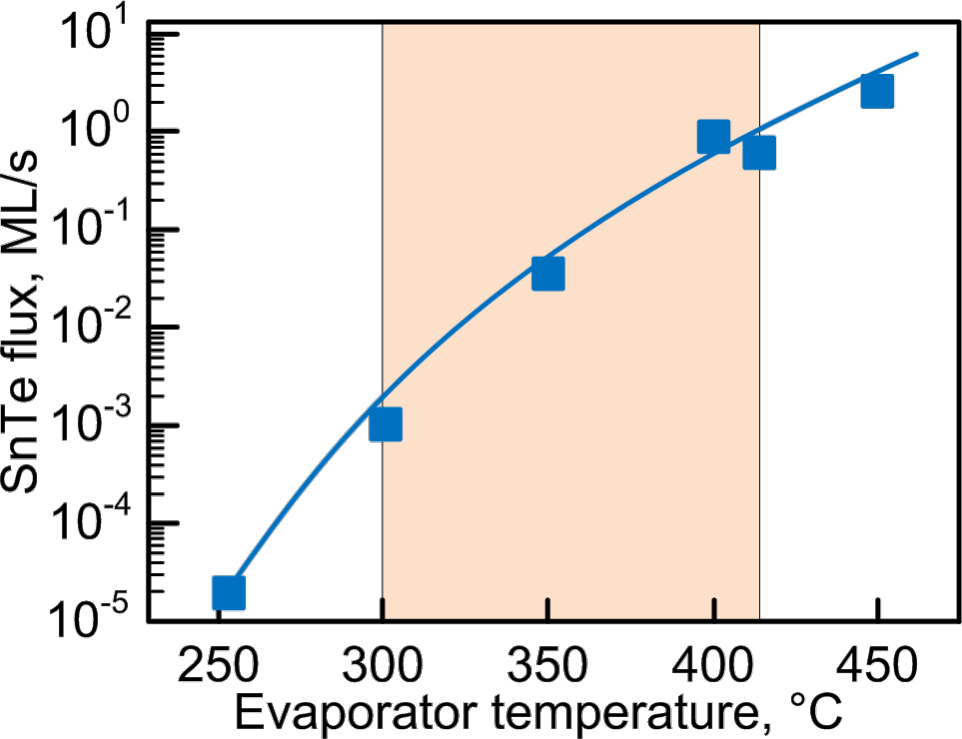
The calibration of the SnTe depositon rate as a function of the evaporator temperature.

The ratio of the fluxes of impurities and growth material is an important problem for the controlled doping during ion beam sputtering [29]. The separation of these fluxes improves the control over the doping process. We proposed a doping method using a solid-state source. Our technique differs from the method described in [30] in so far that only a single ion source used. Instead of a second ion source we used a resistive evaporator. For this purpose, the vacuum chamber was equipped with a 10 mm square graphite evaporator. The usage of such an evaporator does not change the evaporation cosine law. A SnTe solid-state source was used as a dopant. This compound was already used earlier [31–32]. In our case, the usage of elementary tellurium is unacceptable because of its high vapor pressure. The vapor flux from the SnTe source was calibrated in the temperature range of 250–450 °С and recalculated in ML/s (1 ML_SnTe_ corresponds to *f*_1МL_ ≈ 9.9 × 10^14^ cm^−2^). To determine the doping level, samples were made at flux ratios of growth material and impurities *R*_GaAs/SnTe_ in the range from 10^3^ to 10^0^. The dependence of the SnTe deposition rate on the evaporator temperature is given in Figure 2.

The evaporator temperature was varied in the range of 300–415 °С (the marked area in Figure 2) in the experiment. Note that we doped the GaAs spacer layer, not the quantum dots. The temperature range was chosen to balance the rates of components in such a way that the SnTe impurity flux did not exceed the GaAs flux.

The surface morphology was studied with an atomic force microscope Solver HV in the semicontact mode by NSG10 probes using positional marks, which allowed us to identify specific region on a surface [33]. The structure of the quantum dots was studied with a transmission electron microscope Tecnai G2 Spirit. Photoluminescence of the nanostructures was investigated in the spectral range of 1.1 to 1.6 eV at a temperature of 90 K. An injection laser with a wavelength of 402 nm and radiation power of 8.5 mW was used as an optical radiation source. A photoluminescent signal was registered by the monochromator MDR-23 and the cooled germanium p-i-n photo diode.

## Results and Discussion

### Temperature

The temperature dependence of ion beam sputtering was studied under the following conditions. A constant ion beam current of 120 µA was chosen, and the energy of the ions was 150 eV. The angle of incidence of the beam was equal to 50°. The temperature ranged from 450 to 650 °C.

**Figure 3:**
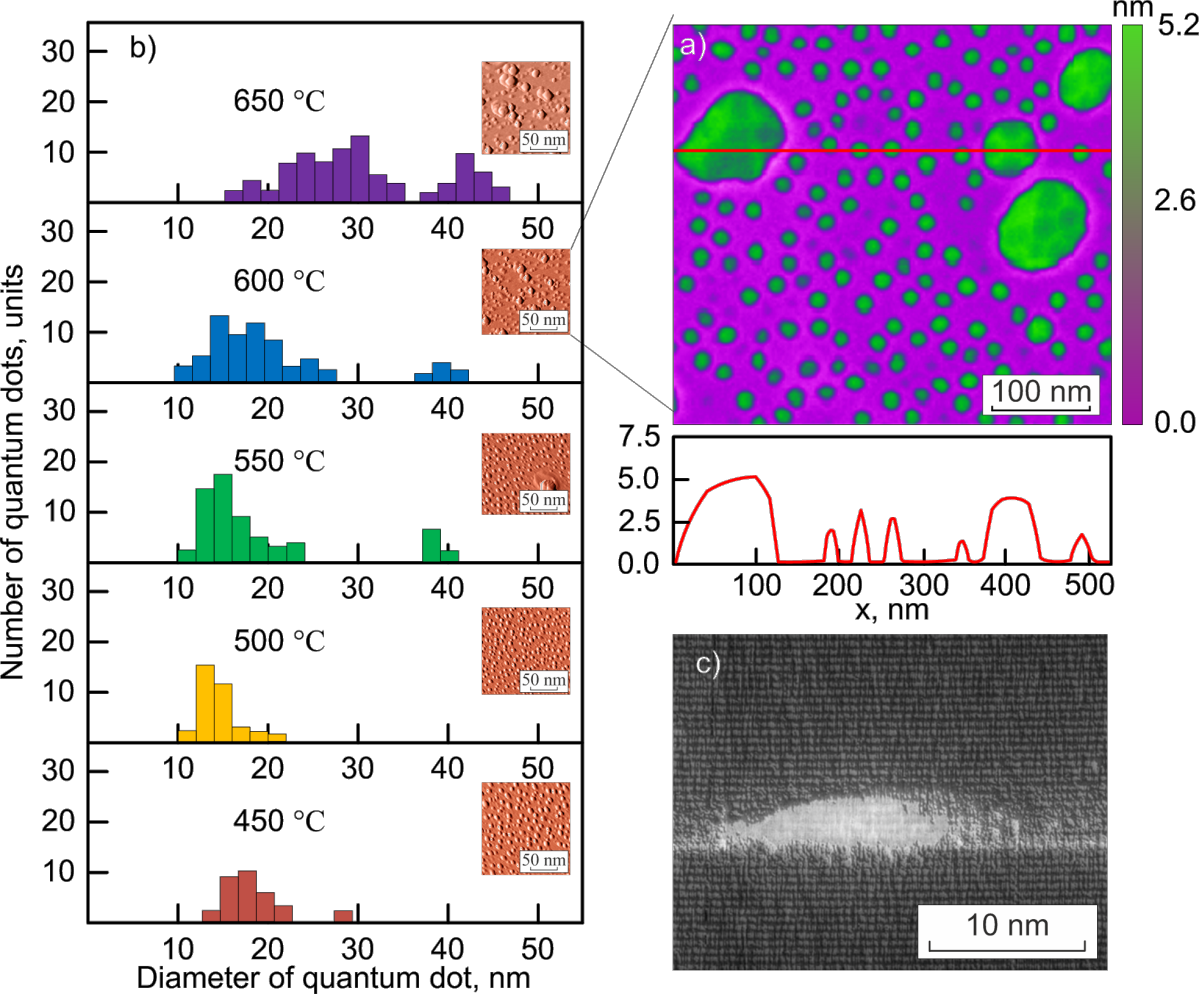
Size distribution of InAs quantum dots as a function of the substrate temperature.

It should be noted that thickness of the InAs wetting layer reaches 1.5–2 ML [34]. InAs/GaAs hut quantum dots contain two extra faces {137} in addition to four main faces {105} [35]. Besides hut-structures, dome dots have four faces {137} and 12 faces with orientation {101} and {111}. This is clearly seen in Figure 3a. The temperature variation reflects on the distribution of the dimensions of InAs quantum dots according to the data given in Figure 3b and Table 1. Figure 3c presents the TEM image of a single hut QD with a clearly seen wetting layer.

**Table 1:** Depending QDs average size and surface density on substrate temperature.

temperature, °C	average size of QDs, nm	surface density, cm^−2^

450	18	0.9·10^11^
500	15	1.1·10^11^
550	17	0.9·10^11^
600	19	0.6·10^11^
650	31	0.4·10^11^

With increasing growth an increase of the size of the quantum dots is observed. The average dimensions of InAs dots at temperatures below 500 °C did not exceed 20 nm. It should be noted that the structural transition from hut to dome at a temperature of 650 °C becomes critical for InAs/GaAs(001). At the same time nanoislands with dimensions from 10 to 50 nm are formed. It can be seen that InAs hut quantum dots can be optimally grown by ion-beam sputtering in a temperature range of 450–500 °C. Increasing of uniformity of the dimension distribution is observed at 500 °C. The dimension dispersion decreases and the linear dimensions are about 15 nm. Separately, we denote the fact of increase in the dimensions of dome structures at the substrate temperatures higher than 550 °С.

The surface density of the nanoislands is also a temperature-dependent parameter (Table 1). Increasing the temperature from 450 to 500 °C practically does not affect the surface density. At temperatures higher than 500 °C, the surface density essentially decreases from 1.1·10^11^ cm^−2^ to 0.4·10^11^ cm^−2^. The existence of the stability regions for indium arsenide (*T* = 450–500 °C) points at the possibility of obtaining quantum dots having acceptably high surface density and small dimensions.

### Ion beam current

**Figure 4:**
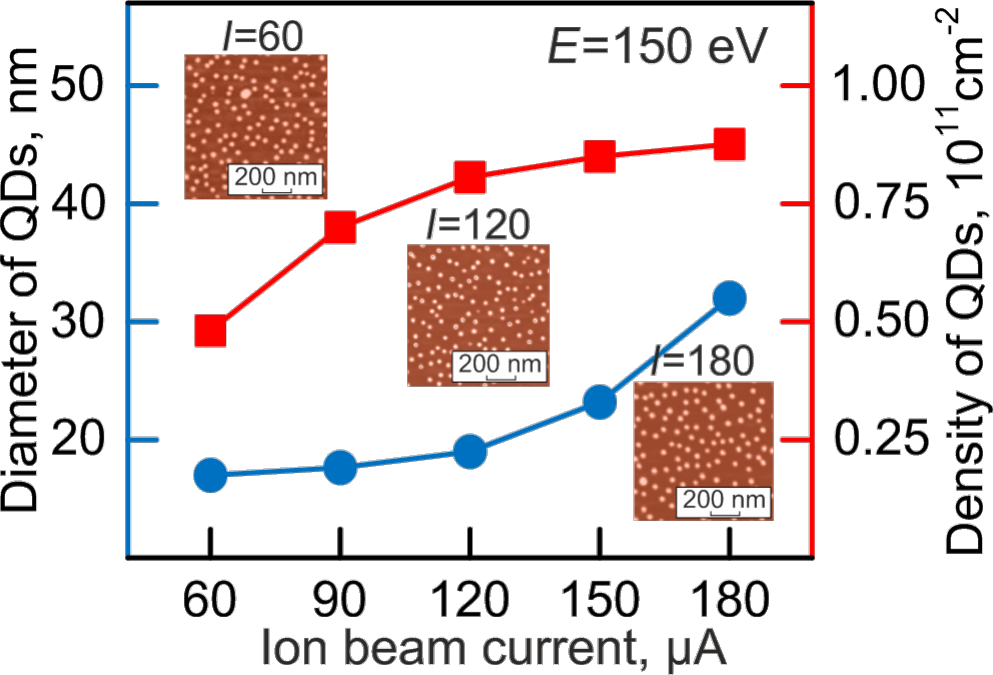
The dependence of average dimensions and surface density of InAs nanoislands on the ion current value.

The value of the ion current can be fundamentally controlled by changing the inlet pressure of the working-gas supply or by varying the voltage of the control electrode. The latter is more preferable because of the higher accuracy of regulation. The influence of ion current value on the island growth was studied under the following conditions. The substrate temperature during indium arsenide crystallization was 500 °C. The ion energy was kept at 150 eV. The ion beam current changed from 60 to 180 µA. The deposition time was chosen in such a way that quasi-layers of the same thickness were obtained in all experiments. As it can be seen in Figure 4 and Table 2, the increase in the ion current is slightly reflected on the value of the average lateral dimensions of InAs/GaAs quantum dots.

**Table 2:** Average size of QDs and surface density as a function of the ion beam current.

ion beam current, µA	QDs average size, nm	surface density, cm^−2^

60	17	0.5·10^11^
90	18	0.7·10^11^
120	19	0.75·10^11^
150	23	0.8·10^11^
180	30	0.85·10^11^

Indium arsenide nanoislands reach average dimensions of about 30 nm at larger currents of 150–180 µA. The observed effect can be explained possibly by the fact that an increase in the ion current causes an increase in the density of mass flow and results in a higher deposition of atoms on the substrate surface at the beginning of and during the growth of quantum dots. These growth conditions are favorable for the formation of more homogeneous islands. The least dispersion of indium arsenide nanoislands is observed when the current values reach 110–120 µA. When the current is further increased, not all incident atoms can be incorporated in the growing quantum dots. Surface diffusion of adatoms appears and is revealed in the non-uniform growth of quantum dots. This effect occurs at currents higher than 150 µA. The smallest uniformity for obtained for hut clusters of indium arsenide was 26%.

It can be seen that the increase in the ion current, first of all, leads to denser arrays of quantum dots. This is confirmed by the AFM images given in the inserts in Figure 4. After comparing the data of the microphotographs and the obtained graphs, firstly it can be seen that it would be necessary to increase the current even further in order to obtain even denser array of nanodots. However, physical and technical reasons impede the realization of this idea. The physical reason consists in the increase in the dimensions of quantum dots and their dispersion, as it was mentioned above. The technical reason consists in that the increase in the current results in an increasingly blurred ion beam profile, and, as a result, in considerable loss of growth material, contamination of the chamber and worsening of the epitaxial conditions. A shorter distance between substrate and target results in a high inhomogeneity of the thickness of the formed quasi-layer. In practice, this results in significant difference in density and dimensions of the islands in the central and peripheral areas of the substrates.

### Ion energy

The energy of the argon ions determines the deposition rate. The synthesis of nanometer-scale layers requires the of the minimum technologically possible deposition rates to avoid sputtering at energies higher than 300 eV. In addition, a nonuniformity of energies of the sputtered atoms is observed in the case of bombarding with high-energy ions.

Basing on the results of the previous two sections, the following conditions were used for studying the influence of the beam energy. The substrate temperature was 500 °C, and a beam current of 120 µA was chosen. The experiments were performed at various energies of the argon ions between 120 eV (lower technical limit of the ion source) and 300 eV.

**Figure 5:**
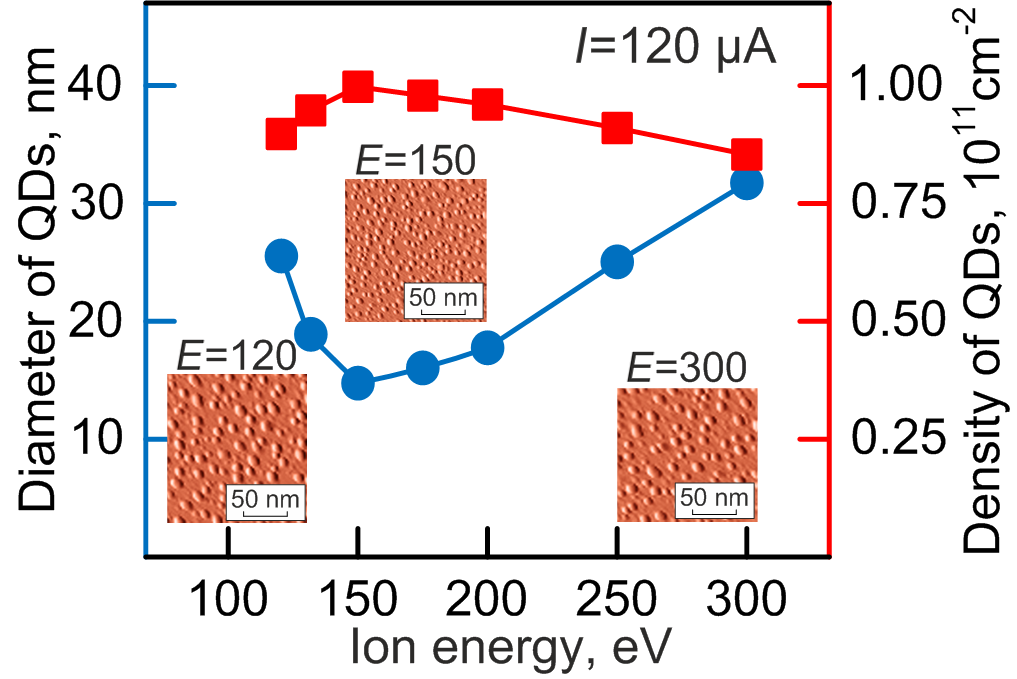
Average dimensions and surface density of InAs nanoislands as a function of the ion energy.

The average lateral dimension of the formed nanoislands of indium arsenide depends non-linearly on the energy of the argon ions as it follows from Figure 5 and Table 3.

**Table 3:** Average size and surface density of QDs as a function of the ion energy.

ion beam current, µA	QDs average size, nm	surface density, cm^−2^

120	27	0.8·10^11^
135	19	0.9·10^11^
150	15	1.1·10^11^
170	16	1.0·10^11^
200	17	0.9·10^11^
250	25	0.85·10^11^
300	31	0.8·10^11^

The smallest dimensions are observed within the energy range of 150–200 eV with quantum dots being smaller than 20 nm. Further increase in the energy leads to an increase in quantum dot size. It is interesting to note that the dimensions of the islands are 27 nm at 120 eV. Apparently, it is caused by a considerable energy dispersion of the primary bombarding ions since the Kauffman ion gun creates a beam that is unstable under monoenergetic conditions at energies near 100 eV. This is caused by the arrangement of net extractors. The wide distribution of the beam energy then causes the size dispersion at 120 eV. Thus, quantum dots with a root-mean-square deviation of the lateral dimensions of more than 50% are formed. This deviation sharply decreases at 150 eV (38% for indium arsenide). At higher energies a slow and almost linear growth of the quantum-dot size dispersion is observed. The value of dispersion did not exceed 50% at the maximum beam energy of 300 eV.

The surface density of nanoislands insignificantly increases with increasing ion energy and reaches 10^11^ cm^−2^. This can be explained by the fact that the density of quantum dots mainly depends on the growth flux, which is determined by the ion current and not the energy. The changes in density correlate with the changes of the sputtering coefficient depending on the ion energy. We note that the values of the ion energy (150 eV) chosen and used in the previous studies were based on the energy patterns determined here.

### Raman spectroscopy

**Figure 6:**
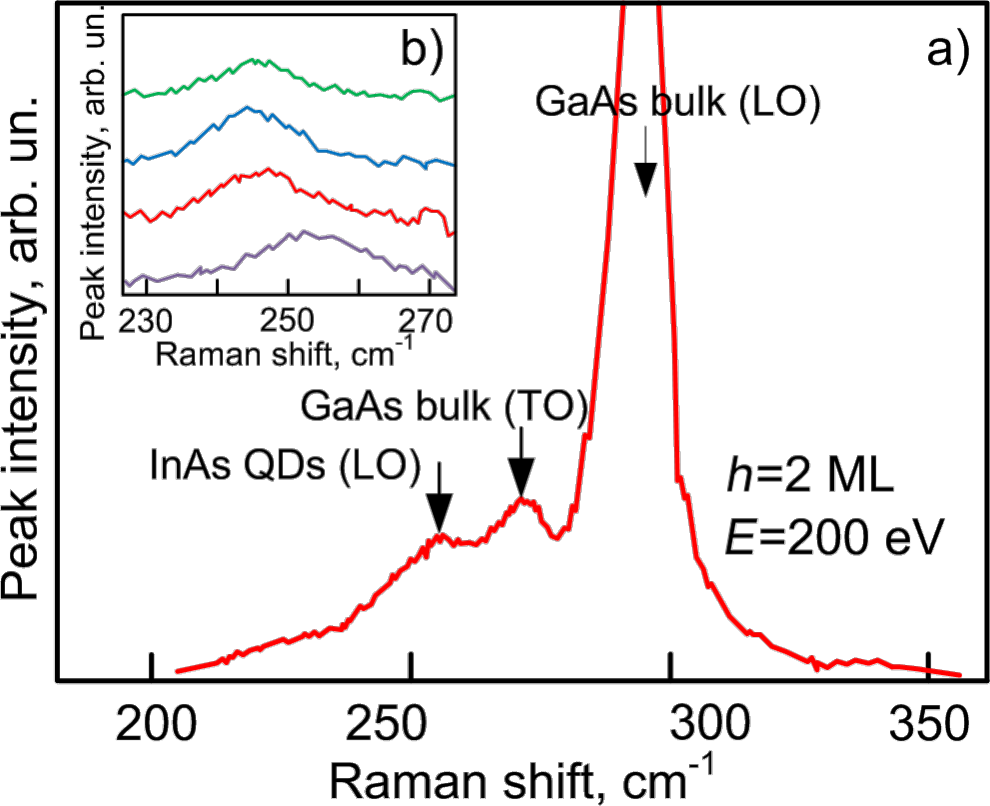
Raman scattering spectra of samples obtained at different ion energies.

Figure 6a shows the Raman spectrum for a sample with a coating layer. There is a high intensity peak at 295 cm^−2^ caused by scattering of LO phonons of the GaAs substrate under *x*(*yz*) polarization. The weak peak at 270 cm^−2^ corresponds to scattering of GaAs TO phonons. To the right, at 254 cm^−2^, there is a peak of Raman scattering of InAs QDs.

Figure 6b shows Raman scattering spectra for samples fabricated at different ion current densities. The shift of the peaks is caused, first of all, by the reduction of elastic stress in the layers with QDs and, then, by an increase in the average QD size. The peaks drift to LO phonon scattering on unstrained single-crystal indium arsenide at 242 cm^−2^. Note that an increase in the QD size is accompanied by a slight inverse shift of the Raman scattering spectrum peak. This is apparently due to strong relaxation of elastic stress in the layer with the dome structures. The obtained Raman scattering data are consistent with the results of microscopy and photoluminescence measurements.

### Concentration profiles

**Figure 7:**
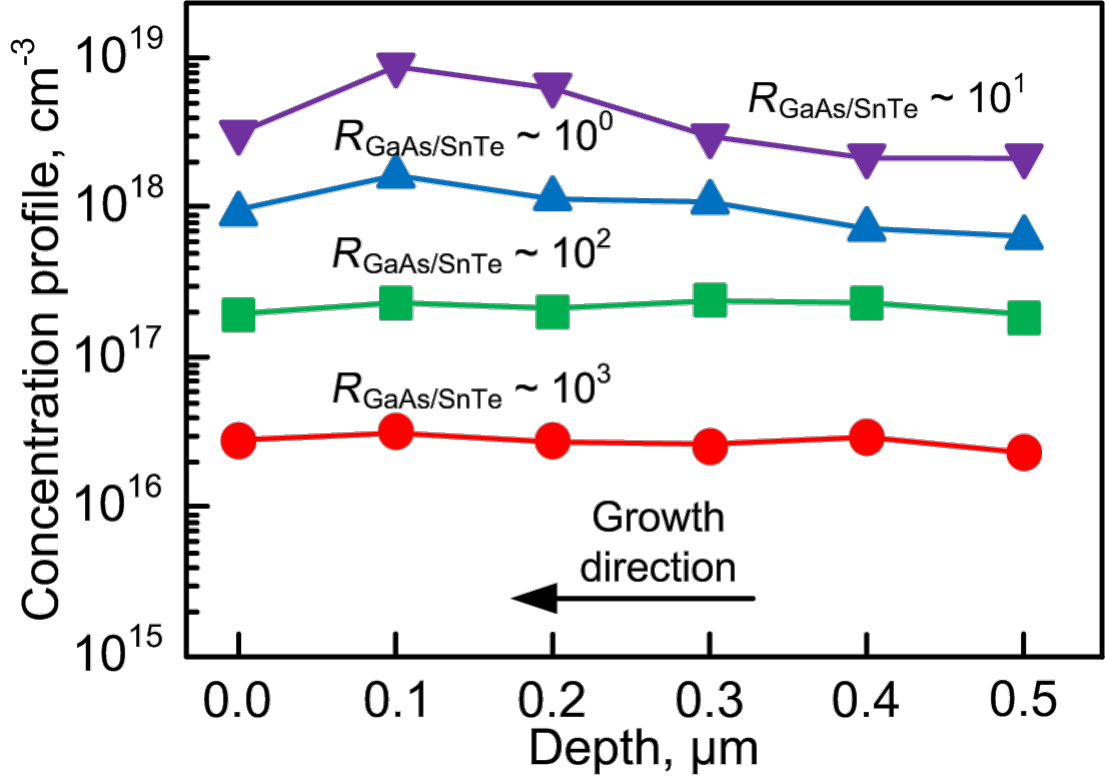
Doping profiles measured by *C*–*V* profiling.

The concentration profiles were obtained by measurement of the capacitance–voltage (*C*–*V*) characteristics using the mercury probe MDC-802B-150 and layer-by-layer chemical etching. All the measured samples showed n-type conduction in the GaAs film. The results are given in Figure 7.

The first doping experiment was carried out at an evaporator temperature of 300 °С and a ratio of flows of *R*_GaAs/SnTe_ ≈ 10^3^. The *C*–*V* measurements showed that the average concentration of charge carriers in the GaAs layer was 2.7·10^16^ cm^−3^. A sufficiently homogeneous distribution of impurities is observed throughout the layer. The increase in the evaporator temperature to 350 °С (*R*_GaAs/SnTe_ ≈ 10^2^) allowed us to reach an average carrier concentration of 2.1·10^17^ cm^−3^. In the doping profile that was obtained at an evaporator temperature of 400 °C (*R*_GaAs/SnTe_ ≈ 10^1^) an increase in impurity concentration along the direction of growth is noticeable. An even more inhomogeneous concentration profile is obtained at 415 °C (*R*_GaAs/SnTe_ ≈ 0^0^). There is a pronounced impurity accumulation along the direction of growth with the concentration fluctuating within one order of magnitude.

The increase in the impurity concentration along the direction of growth for values of *R*_GaAs/SnTe_ between 10^0^ and 10^1^ can be explained by a weakly pronounced impurity segregation [36–38]. Evaporation of the solid SnTe source of can occur in the form of elementary Sn, elementary Te or molecules SnTe [39]. The coefficient of volume diffusion of the impurity components does not change when the gallium arsenide flux and the substrate temperature are kept constant. The decrease of *R*_GaAs/SnTe_ results in a surplus of Te on the growing layer surface. The rate of Te volume diffusion becomes smaller than the growth rate of the GaAs layer resulting in the increase of its concentration in each monolayer. Impurity accumulation becomes visible at *T* > 350 °С when *R*_GaAs/SnTe_ < 10^2^.

We note that the obtained value of the concentration of electrically active donors at the level of 10^18^ cm^−3^ is smaller than impurity concentration corresponding to the SnTe deposition rate. One reason is a systematic error of the doping profile measurement. The used *C*–*V* measurements give information only about the total concentration of donors that were captured by a growing layer surface, passed into its volume by means of diffusion and became electrically active donors. In our case, such donors are Te^+^, Sn^+^ and SnTe^+^ complexes. Obviously, not all evaporated Sn and Te atoms and SnTe complexes are incorporated into the layer volume or are captured by the GaAs surface. The other reason is connected to the physical processes on the growing layer surface. Firstly, a part of the donor atoms can leave the growing layer surface by desorption. Secondly, there is an increasing probability of capturing of impurity atoms by layer defects over the course of growth. Consequently, these impurities cannot be activated. We define the donor activation coefficient, *N*_CV_/*N*_evap_, as the ratio of the measured concentration of charge carriers *N*_CV_ to the amount of evaporated atoms Sn+Te+SnTe in their volume concentration equivalent *N*_evap_ if all of them were captured by the growing layer. This allows us to determine the influence of evaporator temperature on the concentration of electrically active donors at constant substrate temperature and constant growth rate of the GaAs layer. Amphoteric behavior of tin is another probable reason for the discrepancy of concentration of activated donors and injected impurities [40]. It allows tin to occupy Ga vacancies in the course of GaAs layer growth and behave as an acceptor-type impurity partially compensating the concentration of Te^+^ donors. The formation of neutral SnTe complexes results in a decrease in electrically active donors Sn^+^ + Te^+^ incorporated in the GaAs layer. We consider that doping profiles higher than 10^18^ cm^3^ were not obtained as a result of these processes.

### Photoluminescence

**Figure 8:**
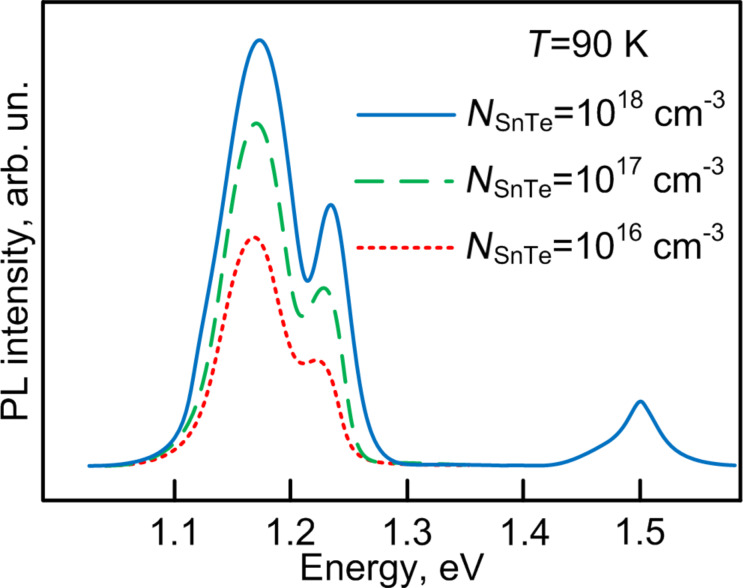
Photoluminescence spectra of the grown InAs/GaAs structures with different levels of doping of the GaAs barrier layer.

The influence of the SnTe doping of the GaAs barrier layer on the photoluminescence of the grown InAs/GaAs nanoheterostructures was studied. The measurements were performed at a temperature of 90 K. The results of these studies are given in Figure 8.

The obtained photoluminescence spectra reflect the ground (*E* = 1.18 eV) and excited (*E* = 1.23 eV) states in InAs quantum dots. The narrow width of the peak at 1.18 eV at half of maximum (FWHM) of radiation corresponds to band-to-band recombination through the ground state of electrons in the conduction band and the valence band. The widening of the peak below FWHM is caused by fluctuation of dimensions of self-organized InAs quantum dots. This value well agrees with the value of energy of the first ground state (1.21 eV) calculated by the 8·k·p method in [41]. The additional peak at 1.23 eV is formed by recombination through levels of excited states in quantum dots. One can see that the increase in SnTe concentration from 10^16^ to 10^18^ cm^−3^ in the GaAs layer results in an increase in intensity of this peak. Electrons generated by the 402 nm laser (ca. 3 eV) become "hot" because of the high excitation energy in the absence of doping in the course of photoluminescence excitation. Under these conditions, "hot electrons" have to be thermalized for participation in radiative recombination according to the zone–zone mechanism. Apparently, such processes are longer in time and affect the photoluminescence intensity. The introduction of impurities creates donor levels in the GaAs band gap and the levels are efficient carrier-capture centers. Owing to this fact, the mechanisms of thermalization and recombination change. The electron lifetime at the donor level is less and an electron is captured more quickly at levels in an InAs quantum dot and participates in radiative recombination acts. This increases the photoluminescence peak intensity.

### Dark *I*–*V* measurements

**Figure 9:**
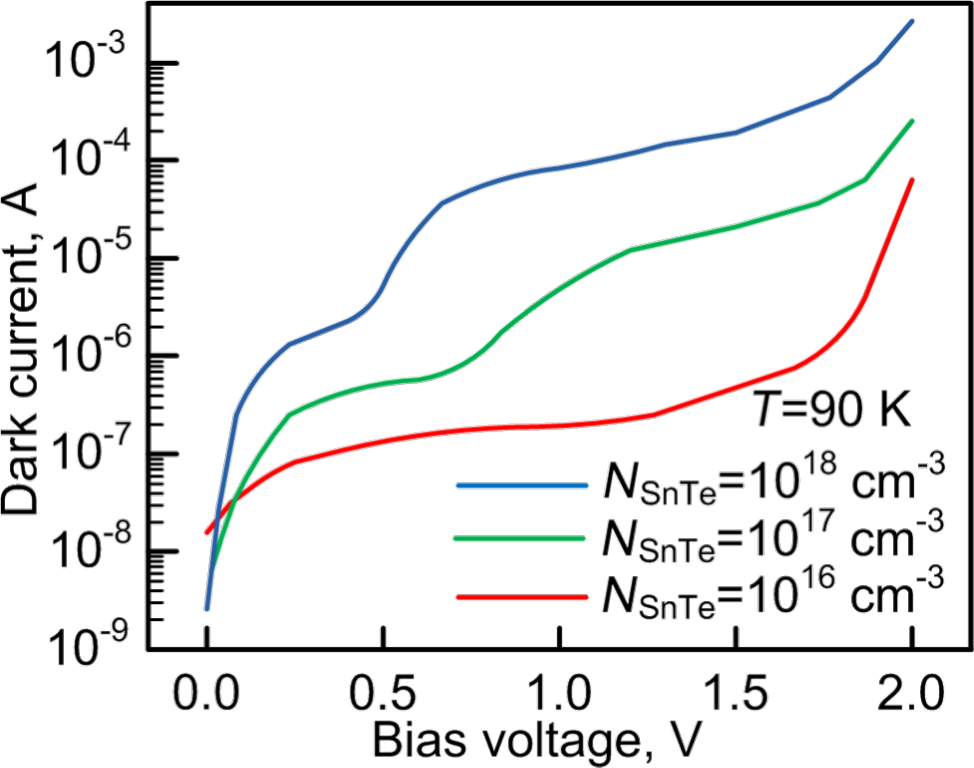
Dark *I*–*V* characteristics of the different doped samples.

It is clear that doping of the spacer layer affects the charge carrier transport from quantum dots into the continuum of the conduction band. However, photoluminescence can only provide information about the energy levels in quantum dots but not about the transport mechanisms. For this purpose, we examined dark current–voltage characteristics of the samples with different doping levels. It is important to note that we doped not the quantum dots but the gallium arsenide spacer layers. Dark *I*–*V* measurements were carried out at a temperature of 90 K. The obtained results were shown in Figure 9.

Two regions can be distinguished in the current–voltage curves. The first region is from 0.0 to 0.5 V, the second region ranges from 0.5 to 2 V. In our opinion the main transport mechanism in region 1 is thermionic emission. In this way charges jump from quantum dot energy levels to the conduction band of the barrier layers. In region 1 the *I*–*V* curves of all samples have saturation regions near 0.5 V. In contrast, sector 2 exhibit different changes in the current with increasing doping level. For a concentration of 10^16^ cm^−3^ a change of the transfer mechanism from thermionic to resonant tunneling can be seen at a bias voltage higher than 1.5 V because of field-assisted tunneling [42]. Simultaneously, the width of the potential barrier is decreasing. Therefore, the tunneling probability through the GaAs barrier layer significantly increases. The main mechanism of carrier transport from InAs QDs becomes a tunneling current. As can be seen from Figure 9 the increase in doping concentration of the barrier layer to 10^18^ cm^−3^ reduces the voltage at which the change of the transport mechanism can been observed to 0.47 V.

## Conclusion

The results of the experimental studies of crystallization of InAs-QD/GaAs(001) quantum-dot nanoheterostructures obtained by ion beam sputtering are presented and analyzed. The average dimension is about 15 nm, the surface density is 10^11^ cm^−2^ in the ion energy range of 150–200 eV at a constant process temperature of 500 °C and a constant beam current of 120 µA. The average dimensions of islands of the both types of materials exceed 30 nm with a dispersion higher than 45% at energies of 300 eV. The controlled doping of the GaAs barrier layer in the InAs-QD/GaAs(001) nanoheterostructure is carried out by ion beam sputtering. A maximum donor concentration of 8.7·10 cm^−3^ is obtained. The effect of impurity accumulation in the direction of layer growth at *R*_GaAs/SnTe_ ≈ 10^0^–10^1^ is explained by segregation that is mainly caused by existence of Te in the ligature source. The presence of impurities in the GaAs barrier layer increases the intensity of photoluminescence peaks of the ground and excited states of the quantum dots. The appearance of donor levels in the GaAs layer promotes faster electron capture at levels in InAs quantum dots and the participation of the electrons in radiative recombination. This increases the photoluminescence peak intensity.
